# Nutritional Quality Analysis and Classification Detection of Buckwheat in Different Harvest Periods

**DOI:** 10.3390/foods13162576

**Published:** 2024-08-17

**Authors:** Peichen Xin, Yun Liu, Lufei Yang, Haoran Yan, Shuai Feng, Decong Zheng

**Affiliations:** 1 College of Agricultural Engineering, Shanxi Agricultural University, Taigu, Jinzhong 030801, China; s20222073@stu.sxau.edu.cn (P.X.); liuyun@sxau.edu.cn (Y.L.); 13467054167@163.com (L.Y.); yhr13466880906@163.com (H.Y.); ds1187948056@163.com (S.F.); 2 Dryland Farm Machinery Key Technology and Equipment Key Laboratory of Shanxi Province, Taigu, Jinzhong 030801, China

**Keywords:** buckwheat, harvest period, nutrient profiling, spectroscopic analysis, category discernment

## Abstract

For buckwheat, the optimal harvest period is difficult to determine—too early or too late a harvest affects the nutritional quality of buckwheat. In this paper, physical and chemical tests are combined with a method using near-infrared spectroscopy nondestructive testing technology to study buckwheat harvest and determine the optimal harvest period. Physical and chemical tests to determine the growth cycle were performed at 83 days, 90 days, 93 days, 96 days, 99 days, and 102 days, in which the buckwheat grain starch, fat, protein, total flavonoid, and total phenol contents were assessed. Spectral images of buckwheat in six different harvest periods were collected using a near-infrared spectral imaging system. Four preprocessing methods (SNV, S-G, DWT, and the normaliz function) and three dimensionality reduction algorithms (IVSO, VCPA, VISSA) were used to process the raw buckwheat spectral data, and the full and eigen spectra were established as a random forest (RF). Random forest (RF) and Least Squares Support Vector Machine (LS-SVM) classification models were used to determine the full and eigen spectra, respectively, and the optimal model for the buckwheat single harvest period was determined and validated. Through physical and chemical tests, it was concluded that the 90-day harvest buckwheat grain protein, fat, and starch contents were the highest, and that the total flavonoid and total phenolic contents were also high. The SNV preprocessing method was the most effective, and the feature bands extracted using the IVSO algorithm were more representative. The IVSO-RF model was the best discriminative model for the classification of buckwheat in different harvest periods, with the correct rates of the training and prediction sets reaching 100% and 96.67%, respectively. When applying the IVSO-RF model to the buckwheat single harvest period to verify the classification, the correct rate of the training set for each harvest period reached 96%, and that of the prediction set reached 100%. Near-infrared spectroscopy combined with the IVSO-RF modeling method for buckwheat harvest period detection is a rapid, nondestructive classification method. When this was combined with physical and chemical analyses, it was determined that a growth cycle of 90 days is the best harvest period for buckwheat. The results of this study can not only improve the quality of buckwheat crops but also be applied to other crops to determine their optimal harvest period.

## 1. Introduction

Buckwheat is a medicinal and food plant rich in nutrients and efficacious substances and has a variety of pharmacological effects. In the field of food processing and medicine, it has considerable potential for development and utilization [1]. The nutrients in buckwheat mainly include starch, protein, fat, flavonoids, and phenols. In health care, buckwheat has antihypertensive, hypoglycemic, hypolipidemic, and antioxidant effects, among other functions [2]. Buckwheat is an indeterminate raceme with a long flowering period. It exhibits side flowering and fruiting, and the seed maturity time and maturity are not consistent [3]. There are many differences in nutrient accumulation in buckwheat grains harvested after different harvest periods. The determination of the optimal harvest period for buckwheat is of great significance to improve the nutritional value of its grains [4]. Near-infrared spectroscopy (NIRS) technology is fast and efficient and is widely used in quality testing, classification, and the identification of various agricultural products [5]. Different buckwheat harvest periods have great impacts on its nutritional quality. They also lead to buckwheat with unique reflectance characteristics in the near-infrared spectral band, and through the analysis of these spectral data, the harvest periods of buckwheat seeds can be identified [6]. Compared with traditional observation and measurement methods, near-infrared spectral imaging technology has higher spectral resolution and accuracy, which can provide more accurate results for harvest period identification. At the same time, this reduces the manpower and time costs, as the use of near-infrared spectroscopy can be quickly applied for the classification of different buckwheat harvest periods.

To date, researchers have carried out significant research work in two areas: nutritional quality and grain classification and detection in different harvest periods. In terms of nutritional quality, the current research is mainly focused on wheat, corn, and other major grain crops; however, there is no research on buckwheat during different harvest periods. Gong, Li, and Lu [7] studied the impact of different harvest times on the nutritional flavor quality and starch RVA characteristics of “Japonica 46”. They considered how delaying the harvest time of “Japonica 46” affected the protein content, the straight-chain amylose content, and rice disintegration. It was found that delaying the harvesting time had less of an effect on the protein content and straight-chain starch content of “Japonica 46”, while the disintegration and abatement values of rice were more affected by the harvesting time. Wang et al. [8] studied the effect of the variety and the harvesting period on the biological yield and silage quality of whole maize and found that with a delay in the silage period, the contents of crude protein, starch, acetic acid, and lactic acid gradually decreased, the content of fat and water-soluble carbohydrates first rose and then declined, and the PH value first decreased and then increased. Liang et al. [9] analyzed the effect of the harvesting period on the yield and nutrient composition of oats and found that the dry matter content gradually increased with delays in the fertility period and that the difference was significant. The dry matter yield in the milky stage was higher than that in the tasseling and grouting stages. In addition to the crude fat, the crude protein, crude ash, and the content of calcium and phosphorus were all reduced. The main focus in classification detection is on grain variety identification, pest and disease monitoring, and seed vigor prediction. Wu et al. [6] collected spectral images of 27 types of sorghum varieties using a hyperspectral imaging system and utilized CARS for data dimensionality reduction. The correct rates of the CARS-RF model training set and the prediction set reached 95.00% and 84.07%, respectively. Liu et al. [10] proposed an algorithm for the rapid visual recognition of a large number of wheat blast seed samples employing images collected using a hyperspectral imaging system in combination with machine learning. This showed good recognition results, with the accuracy of the modeling and test sets reaching 95.5% and 98%, respectively. Larios et al. [11] proposed an algorithm for the rapid visual recognition of a large number of wheat blast seed samples using images collected via a hyperspectral imaging system combined with chemometric methods; they used a PCA method for data dimensionality reduction combined with support vector machine (SVM), K-nearest neighbor (KNN), and discriminant analysis to differentiate soybean seed vigor, and were able to differentiate 100% of soybean seed vigor levels. Yang, You, and Cheng [12] used hyperspectral imaging to study the spectral characteristics of corn seeds with different maturity levels. The feature band images were processed using image analysis methods to identify corn seeds with lower maturity levels, and the average correct identification rate was 93.9%. Singh et al. [13] collected 35 barley varieties, and hyperspectral reflectance images of the ventral and dorsal sides of the seeds were captured in the near-infrared (NIR) range from 900 to 1700 nm. The average spectra were extracted and preprocessed using a variety of methods, and NIR images of the ventral and dorsal sides of the seeds were collected using a variety of methods. Extraction, preprocessing, and NIR-HSI were combined with an end-to-end CNN, demonstrating accuracy greater than 98%.

In summary, in terms of nutritional quality, current research is mainly focused on corn, wheat, and other major grain crops; research on buckwheat is relatively rare. In terms of grain classification and detection, research on buckwheat during different harvesting periods has not yet been undertaken. Nutritional quality research within grain classification and detection comprises two elements; this study uses physicochemical tests to analyze the nutrient quality of buckwheat in different harvesting periods and near-infrared spectroscopy nondestructive testing technology to determine different harvesting periods for buckwheat classification detection so as to determine buckwheat’s optimal harvesting period.

## 2. Materials and Methods

### 2.1. Test Materials

The buckwheat samples selected for this study are red mountain buckwheat from the Shen Feng experimental field at Shanxi Agricultural University, Taigu District, Jinzhong City, Shanxi Province (37°25.6′ N,12°35.0′ E, temperate continental climate throughout the year); the soil in the experimental field is sandy. The average annual rainfall is 391 mm and the average temperature is 13 °C. Red mountain buckwheat was planted on 1 July 2023 following a planting method for mechanical strip sowing; there was a sowing spacing of 50 cm and a sowing density of 2,100,000 plants/hectare. Buckwheat varieties with good growth were chosen, and full buckwheat grain was chosen. The buckwheat harvest did not fail, there were no weeds in the field, and there was almost no natural grain. In this study, buckwheat was divided into six harvest periods: the growth cycles were 83 days, 90 days, 93 days, 96 days, 99 days, and 102 days. Some of the buckwheat was manually threshed on the day of the harvest and brought back to the laboratory immediately after harvesting. We selected buckwheat kernels with a uniform shape and size, no cracks, fuller shapes, and normal colors as test samples. We used a Starter Kit visible–near-infrared hyperspectral (Headwall photonics, Bolton, MA, USA) scanning platform on the same day to take images of buckwheat samples in different periods.

### 2.2. NIR Spectral Imaging Acquisition Equipment

**Figure 1:**
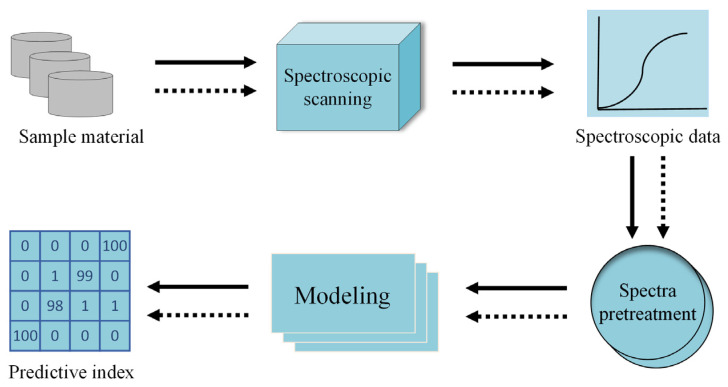
Spectral analysis flow chart.

The system consisted of a scanning platform, a miniature near-infrared spectral imager (aperture 1.4, focal length 25 mm), a light source, a controller, a computer, etc. It had a spectral range of 900–1700 nm, a spectral resolution of 0.727 nm, and an effective wavelength band of 950–1650 nm. The acquisition parameters were set as follows to obtain clear and undistorted images: object distance, 370 mm; push-sweep stroke, 100 mm; and platform moving speed, 2.938 mm. The buckwheat seed samples were loaded into a sample dish with a diameter of 40 mm and a depth of 15 mm, smoothed and compacted, and placed on the mobile scanning platform for near-infrared spectral image acquisition. Each harvest sample was imaged 100 times, totaling 600 NIR spectral images. A flow chart for the spectral analysis is shown in Figure 1.

### 2.3. Determination of Nutritional Quality of Buckwheat during Different Harvest Periods

After collecting NIR spectral information, buckwheat samples were used for the determination of physicochemical properties after growth cycles of 83, 90, 93, 96, 99, and 102 days. We used acid hydrolysis to detect the starch content of the sample [14], Kjeldahl nitrogen determination to detect the protein content of the sample [15], Soxhlet extraction to detect the fat content of the sample [16], sodium nitrate aluminum nitrate to determine the total flavonoid content of the sample [17], and the Folin–Ciocalteu colorimetric assay to detect the total phenol content of the sample [18]. All of the experiments were replicated three times. All of the tests were repeated three times.

### 2.4. Extraction and Processing of Near-Infrared Spectral Data for Buckwheat in Different Harvest Periods

#### 2.4.1. Data Extraction

NIR spectral images contain both spectral and image information of the buckwheat sample, and each pixel point on the image has a diffuse reflectance spectral curve corresponding to it. In this study, ENVI 5.0 [19] was used to extract the spectral data from the region of interest (ROI) of each hyperspectral image. The reflectance of each pixel point was calculated and the arithmetic mean was taken, which was used to form the basic data set for subsequent data processing.

#### 2.4.2. Preprocessing of Near-Infrared Spectral Data for Buckwheat in Different Harvest Periods

During the acquisition process, the noise of the instrument itself as well as the surrounding stray light affect the spectral information. To attenuate or eliminate this effect, the collected spectral data need to be preprocessed to improve the accuracy and stability of model detection. In this study, four preprocessing methods, namely Standard Normalized Variate [20,21] (SNV), Discrete Wavelet Transform [22] (DWT), normalization [23], and Filter Smoothing [24] (Savitzky–Golay, S-G), were used to preprocess the original spectral data, respectively. They were combined with the classification algorithm to compare the correct rate of both the training and prediction sets, and the preprocessing method with the best effect was selected. The reasons for choosing these four preprocessing methods are as follows: SNV preprocessing can significantly reduce interference due to factors such as scattering effects and baseline drift in spectral data; DWT can effectively separate noise and background in spectral signals and improve the signal-to-noise ratio of the spectral data; normalization helps to eliminate the magnitude differences between different samples or bands, making the data more uniform and comparable; and S-G filtering can smooth the spectral curve while retaining more spectral details. Considering the stability and general adaptability of the constructed model, the total number of samples in the modeling part was 600 and the training and prediction sets were randomly divided in a 3:1 ratio; the training set contained 450 samples and the prediction set contained 150 samples.

#### 2.4.3. Extraction of Characteristic Wavelengths from Near-Infrared Spectral Data of Buckwheat in Different Harvest Periods

In the original full-spectrum data, a large amount of redundant and covariance data was included, which significantly affects the model’s accuracy and data processing efficiency. Therefore, it was necessary to extract and analyze closely related wavelengths, simplify the model, eliminate useless information, and obtain a model with strong predictive ability and good stability.

In this study, three methods, IVSO, VCPA, and IVSSO, were used to extract the characteristic wavelengths from the preprocessed spectral data.

The iterative variable subset optimization (IVSO) [25] algorithm gradually eliminates informative variables by employing the WBMS strategy and sequential summation with the regression coefficients of PLS as the importance of the variables. Variable combination cluster analysis (VCPA) [26] is a high-performance variable selection algorithm. It first tests the spectrum of the sample using Binary Matrix Sampling (BMS) and then calculates the frequency of each variable in the optimal sub-model using the concept of model cluster analysis (MPA). The importance of the variables is determined based on frequency, and an exponential decreasing function is applied to eliminate the less influential variables. The variable iterative space shrinkage approach (VISSA) [27] algorithm is a kind of model cluster analysis (MPA) based on the idea of gradually optimizing the variable space during each iteration, ultimately achieving selection of the optimal combination of variables.

#### 2.4.4. Classification Models and Assessment Indicators

The random forest algorithm predicts the outcome by constructing multiple decision trees and voting, and usually achieves a high prediction accuracy. LS-SVM is an algorithm that improves on traditional support vector machines (SVMs) by solving a system of linear equations instead of the quadratic programming problem in traditional SVMs, which simplifies the computational process and improves learning efficiency. These two algorithms have significant advantages in solving specific problems. Thus, we used random forest and Least Squares Support Vector Machine classification models.

The random forest (RF) [28,29] algorithm, which is currently widely used in regression and classification problems, is an intelligent combinatorial classification algorithm that uses a self-service sampling method (bootstrap) for the original training sample set, N, in the put back to randomly sample n samples and generate a new set of training samples to train the decision tree. It repeats the above steps to generate m decision trees and form a random forest; the classification result of the new data is based on the score formed by the number of votes the classification tree has. The classification ability of a single tree may be small, but after randomly generating a large number of decision trees, a test sample can be statistically selected using the classification results of each tree for the most likely classification.

Least Squares Support Vector Machine (LS-SVM) [30,31] is a classification algorithm widely used in fault diagnosis. The basic premise of SVM is to map the data into a high-dimensional space and then find a hyperplane in the high-dimensional space to separate the samples of different classes. LS-SVM is a variant of SVM which simplifies the solution process by introducing the kernel function to convert the nonlinear problem into a linear problem. LS-SVM is an improved support vector machine proposed to reduce training time and computational complexity and improve generalization ability. The radial basis function was chosen as the kernel function of the LS-SVM model to reduce computational complexity by solving a system of linear equations instead of the complex quadratic programming problem characteristic of SVM. LS-SVM has been used for the qualitative and quantitative analysis of spectra.

These two classification algorithms were combined with three feature band extraction methods to obtain six classification models: IVSO-RF; VCPA-RF; VISSA-RF; IVSO-LS-SVM; VCPA-LS-SVM; and VISSA-LS-SVM. The optimal models obtained from the comparisons were then validated for the classification of a single harvest period.

These six classification models were evaluated in terms of accuracy, error rate, precision, recall, and F1 score. Accuracy is the most intuitive performance metric; it indicates the proportion of correctly classified samples to the total number of samples. The error rate is the proportion of the number of incorrectly classified samples to the total number of samples. Precision indicates the proportion of all samples that are predicted by the model as positive that are truly positive. Recall, also known as the true instance rate, indicates the proportion of all samples that are truly positive that are correctly predicted to be positive by the model. The F1 score is a reconciled average of precision and recall and is used to demonstrate the performance of the model in terms of both precision and recall. The formulas for accuracy, error rate, precision, recall, and F1 score are as follows:

(1) $$\mathrm{Accuracy}=\frac{\mathrm{TP}+\mathrm{TN}}{\mathrm{TP}+\mathrm{FP}+\mathrm{FN}+\mathrm{TN}}$$

(2) $$\mathrm{Error}\;\mathrm{Rate}=\frac{\mathrm{FP}+\mathrm{FN}}{\mathrm{TP}+\mathrm{FP}+\mathrm{TN}+\mathrm{FN}}$$

(3) $$\mathrm{Precision}=\frac{\mathrm{TP}}{\mathrm{TP}+\mathrm{FP}}$$

(4) $$\mathrm{Recall}=\frac{\mathrm{TP}}{\mathrm{TP}+\mathrm{FN}}$$

(5) $$F1\;\mathrm{Score}=\frac{2\times \mathrm{Precision}\times \mathrm{Recall}}{\mathrm{Precision}+\mathrm{Recall}}$$

In the formulas, true positive (TP) represents the number of true samples that are identified as true; false negative (FN) represents the number of true samples that are identified as false; false positive (FP) represents the number of false samples that are identified as true; and true negative (TN) represents the number of false samples that are identified as false. A higher accuracy rate indicates a better discriminative effect and the error rate is complementary to the accuracy rate. A higher precision indicates a higher proportion of the samples predicted to be positive that are truly positive, while a higher recall means that more truly positive cases are found by the model. The F1 score is used to comprehensively reflect the performance of the model in terms of precision and recall.

## 3. Results and Analysis

### 3.1. Analysis of Nutritional Quality Results for Buckwheat in Different Harvest Periods

**Figure 2:**
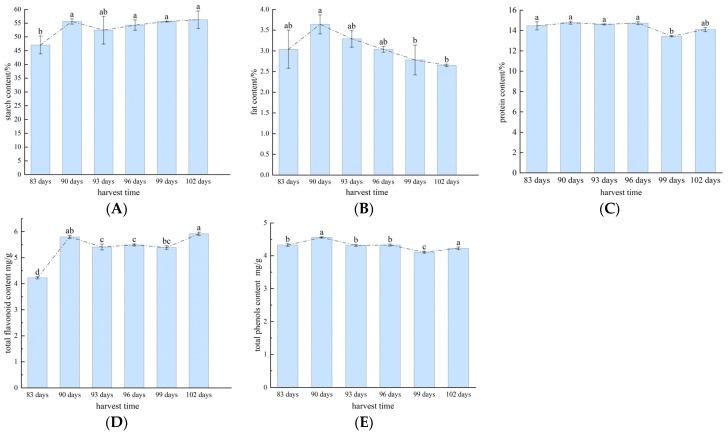
Changes in the content of essential nutrients: (**A**) starch content; (**B**) fat content; (**C**) protein content; (**D**) total flavonoid content; (**E**) total phenol content. Different letters (a, b, c, d) indicate significant differences.

The changes in the starch, fat, protein, total flavonoid, and total phenol contents of buckwheat in different harvest periods are shown in Figure 2.

Starch was higher throughout the harvest period for the 90- and 102-day harvests and lowest for the 83-day harvest. The starch content increased substantially from the 83rd day to the 90th day but decreased from the 90th day to the 93rd day; from the 93rd day onwards, the starch content gradually increased without substantial changes.

Fat had the highest content of 3.64% after 90 days; from the 90th day, the fat content gradually decreased, and in the 102-day harvest, buckwheat fat content was the lowest at 2.65%.

Protein content remained stable throughout the harvest period, ranging from 13.43 to 14.76%. The buckwheat protein content after 90 days was slightly higher than the other periods, with a value of 14.76%.

The total flavonoid content showed a trend of increasing, then decreasing, and then increasing. It was lowest at 4.23 mg/g in the 83-day harvest and increased considerably to 5.8 mg/g from the 83rd day to the 90th day, but then decreased from day 90 to day 93 and increased slightly in the 102-day harvest.

The variation in the total phenol content was consistent with that of total flavonoids. The 83-day harvest had the lowest total phenol content at 4.33 mg/g; the 90-day harvest had a higher total phenol content of 4.56 mg/g.

The results for the nutritional quality of buckwheat in different harvest periods are shown in Table 1. The coefficient of variation of fat and total flavonoids was significantly higher than that of the other components, reaching 11.60% and 11.18%, respectively. The coefficient of variation of starch was also higher at 6.43%. This indicates that different harvest periods led to large differences in the content of these three components in the harvested buckwheat. The coefficients of variation for protein and total phenol were relatively low, at 3.56% and 3.43%, respectively. Comprehensive analysis shows that the content of starch, protein, fat, total flavonoids, and total phenols changes and that buckwheat has an optimal harvest period of 90 days.

### 3.2. Analysis of Near-Infrared Spectral Results of Buckwheat in Different Harvest Periods

#### 3.2.1. Spectral Characterization

**Figure 3:**
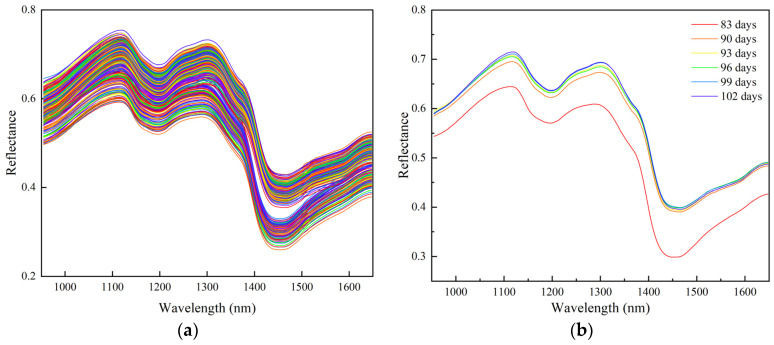
Buckwheat spectral curve: (**a**) original spectral curve; (**b**) average spectrum.

The original spectral curves of the 600 samples selected for this experiment are shown in Figure 3a. The average spectral curves from six different buckwheat harvest periods are shown in Figure 3b. In the wavelength range of 950~1650 nm, the overall trend in the buckwheat spectral curve is consistent; peaks and troughs are evident and some of the curves appear to be overlapping. Further analysis shows that the two peaks are located at 1150 nm and 1303 nm, and the two troughs are located at 1203 nm and 1460 nm. The 1150 nm peak and 1203 nm trough may be caused by C-H secondary frequency doubling [32,33], the 1303 nm peak may be related to C-H secondary frequency doubling [33], and the trough at 1460 nm may be caused by N-H telescopic vibration [34]. However, due to the differences in the content and proportion of chemicals contained in buckwheat kernels in different harvest periods, there are differences in the reflectance of buckwheat kernels. These differences provide an effective discriminatory basis for the spectral identification of buckwheat kernels from different harvest periods.

#### 3.2.2. Analysis of Data Preprocessing Results

**Figure 4:**
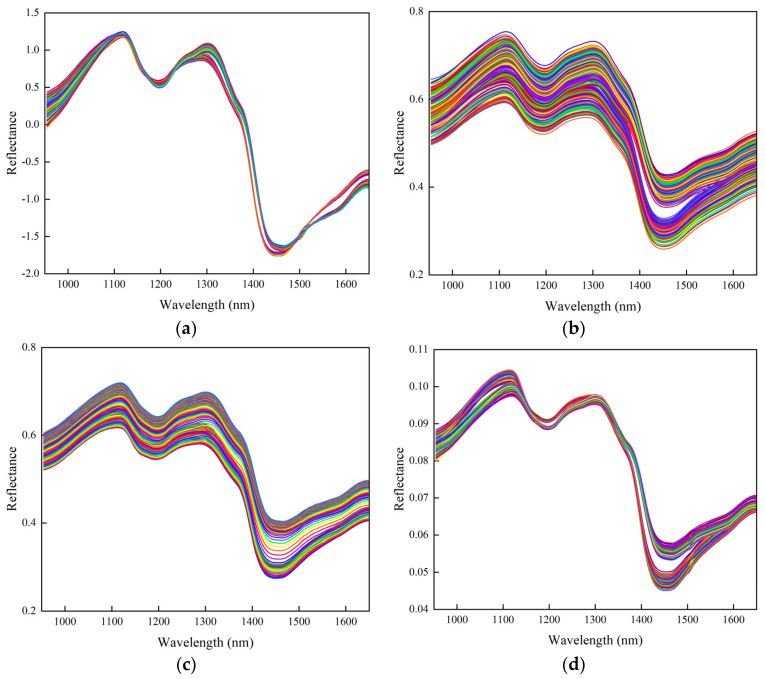
Pre-processed buckwheat spectra: (**a**) SNV−treated; (**b**) S-G-treated; (**c**) DWT-treated; (**d**) normaliz-treated.

SNV, S-G, DWT, and normaliz were selected to preprocess the buckwheat spectra; the preprocessed spectral curve is shown in Figure 4. The preprocessed curve is smoother and the noise and fluctuations in the original data are reduced, making the trend more clear, which is conducive to improving the classification accuracy of the model.

The results of the constructed classification models based on the data from the four preprocessed spectral methods, SNV, S-G, DWT, and normaliz combined with RF and LS-SVM algorithms, are shown in Table 2. SNV preprocessing leads to the highest correctness rate for both the training and prediction sets. Therefore, the SNV preprocessing method is selected.

#### 3.2.3. Analysis of Feature Wavelength Extraction Results

The characteristic wavelengths were extracted from the SNV preprocessed spectral data using three methods: IVSO, VCPA, and VISSA.

**Figure 5:**
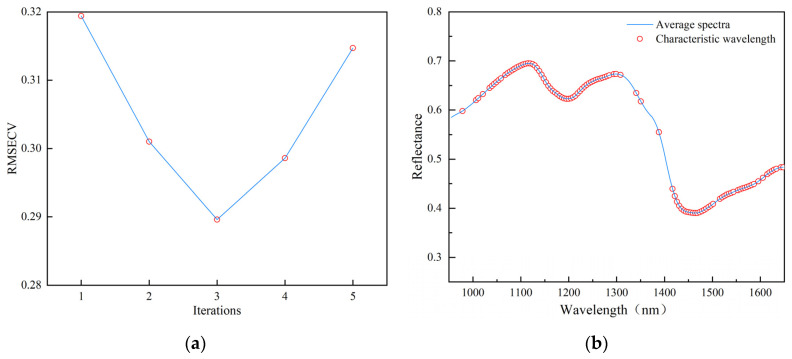
IVSO-based feature wavelength extraction: (**a**) plot of variation in RMSECV values in each iteration; (**b**) distribution of characteristic wavelengths over the full spectrum.

The curve of the RMSECV of the PLS model with the number of iterations during the operation of IVSO is shown in Figure 5a. It can be seen that the value of RMSECV decreases after the first two iterations and then shows a gradual upward trend from the third iteration. This is due to the fact that in the first two IVSO iterations, a large number of irrelevant and interfering variables are gradually eliminated, while in the third iteration, due to the reduction in the number of variables, some useful variables are eliminated and thus the RMSECV value of the model rises. Therefore, a minimum RMSECV value of 0.2896 was found in the third iteration, and the 106 wavelength variables obtained from the final IVSO calculation were the feature variables, as shown in Figure 5b. This reduced the spectral wavelengths to 71.62% of the full spectrum, and these feature variables were mainly distributed in the 1050–1300 nm and 1400–1650 nm regions, concentrated near the first peak and two troughs.

**Figure 6:**
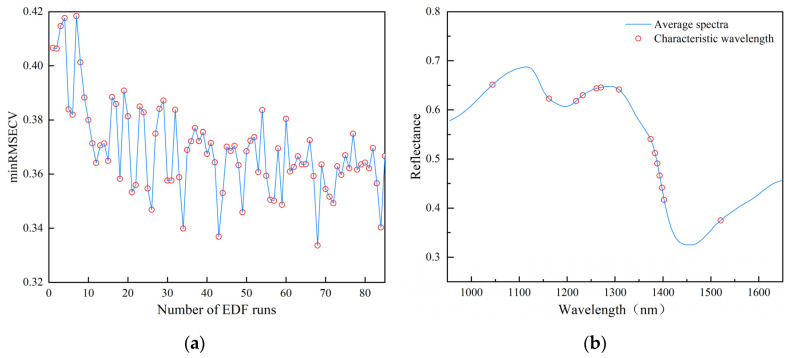
VCPA-based feature wavelength extraction: (**a**) graph of variation in minRMSECV; (**b**) distribution of feature wavelengths over the full spectrum.

The process of extracting feature variables based on the VCPA method is shown in Figure 6a. With the increase in the number of EDF runs, the minRMSECV value decreases overall, which indicates that the covariates in the original data are being gradually screened out and the predictive performance of the model is gradually improving. After the 43rd run, the minRMSECV value decreases sharply and the error starts to increase after the continuation of the run. When ran 68 times, the minRMSECV value reaches a minimum of 0.3336, and the best combination of feature variables is obtained. Only 14 feature variables were extracted using the VCPA method, as shown in Figure 6b. This only accounted for 9.46% of the original spectra, and the feature variables near the first peak and the two valleys, which were mainly distributed in the 1044 nm, 1162 nm, 1230 nm, 1270 nm, 1383–1402 nm, and 1502 nm regions, were mainly excluded.

**Figure 7:**
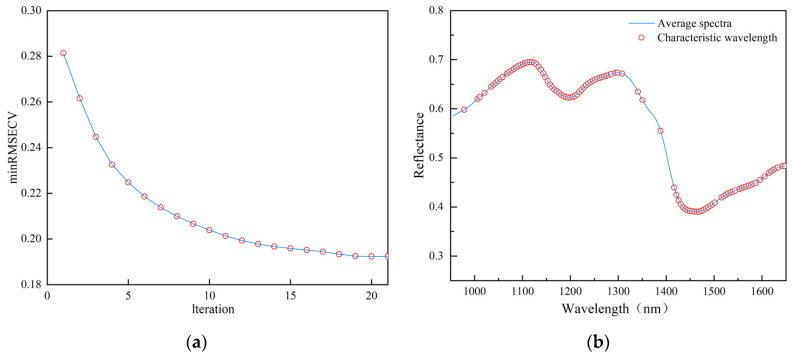
VISSA-based feature wavelength extraction: (**a**) graph of the variation in minRMSECV; (**b**) distribution of feature wavelengths over the full spectrum.

The VISSA method is based on the idea of model cluster analysis. It gradually optimizes the variable space in each iteration and obtains the feature variables by continuously reducing the root mean square error of cross-validation (RMSECV). Figure 7a shows the change in the minimum error value of the model during the iteration process. During this process, the minimum error value gradually decreases from the initial 0.2814, indicating that redundant variables are eliminated in each iteration and that the prediction accuracy of the model is gradually improving. After the 21st iteration, the minRMSECV value reaches a minimum of 0.1923, at which time 77 feature variables are obtained, as shown in Figure 7b, accounting for 52.03% of the original number. They are mainly distributed in the 1050–1300 nm and 1410–1650 nm regions, concentrated in the vicinity of the first wave peak and two wave valleys.

#### 3.2.4. Analysis of Classification Results

First, RF and LS-SVM classification models were established for full-spectrum buckwheat data. The training and prediction sets’ correctness rates for the LS-SVM classification model built on raw spectral data of buckwheat from different harvest periods were low, at 39.79% and 35.33%, respectively; the training and prediction sets’ correctness rates for the RF classification model were high, at 100% and 73.33%, respectively. After preprocessing using the SNV method, the prediction set correctness rate of the LS-SVM model reached 64.67%, and that of the RF model was 92%. Therefore, the RF classification prediction model was finally selected.

The results of the combined RF and LS-SVM classification algorithms with IVSO, VCPA, and VISSA—the three feature band extraction methods—are shown in Table 3. The IVSO downscaling method, which extracts the feature bands, is more accurate and more representative. The accuracy of buckwheat harvesting period prediction is the highest for the IVSO-RF model with 500 spanning trees (ntree) and 10 features selected with random sampling (mtry); the prediction set accuracy and F1 score reached 96.67% and 92.58%, respectively. The overall correctness of the IVSO-RF classification model for the prediction set is significantly better than that of the other five models. Therefore, the IVSO-RF model was selected as the optimal model for predicting different buckwheat harvest periods.

**Figure 8:**
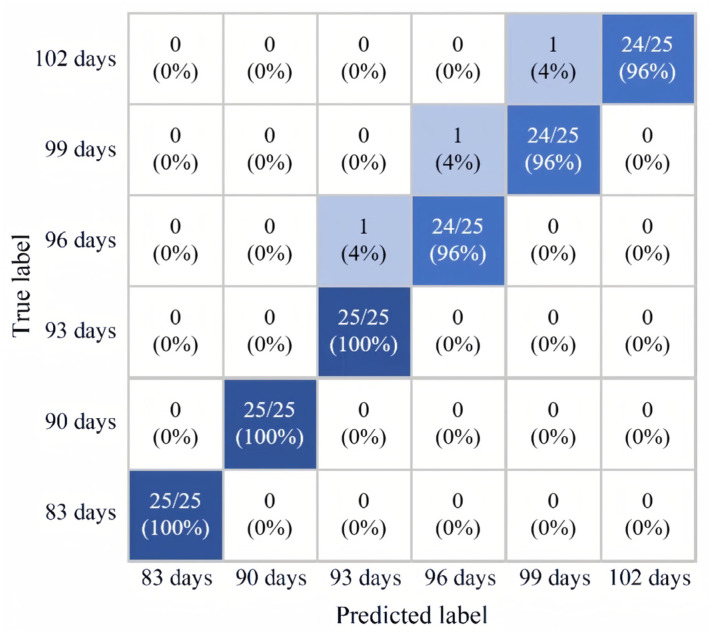
IVSO-RF model confusion matrix diagram.

The results of classification validation for which we applied the IVSO-RF classification model to a single harvest period of buckwheat are shown in Figure 8, the more intense the color, the higher the level of classification. With the correctness rates of the training and prediction sets for each harvest period reaching above 96% and 100%, respectively.

## 4. Discussion

The nutritional quality of buckwheat changes with different harvesting periods, although physical and chemical tests to determine the starch, fat, protein, total flavonoid, and total phenolic contents can determine trends [35,36]. In the buckwheat ripening process, starch content first rises, then falls and rises again, with an overall upward trend [37,38]. This is likely because, in the early stages, the rate of starch synthesis exceeds the rate of starch decomposition, resulting in an increase in starch content. With the passage of time, the starch content decreases when the decomposition rate increases or the synthesis rate decreases, and then the starch content increases a second time when the starch synthesis rate exceeds the decomposition rate due to external environmental factors. The fat content showed a trend of increasing and then decreasing, probably because lipids are transported to the seed to support seed growth and development in the seed maturation process; as the seed matures, lipids gradually become used to synthesizing other biomolecules, resulting in a decrease in fat content. This is consistent with the results of Wang [8]. The protein content of buckwheat in different harvest periods did not change much and remained stable, which is consistent with the results of Fan [39]. In the early stages of maturation, buckwheat metabolism is active and functional components may be produced, and thus they accumulate; then, as buckwheat plants begin to enter the senescence stage, metabolism slows down, the synthesis of functional components is reduced, and it may begin to decrease. Functional components may be used in the later stages of maturation, and the protective mechanism activity may increase again. The total flavonoid and phenol contents showed a trend of first increasing, then decreasing and increasing again, which was consistent with the results of Cheng [18].

Through the IVSO algorithm, 106 characteristic bands were screened for buckwheat in different harvest periods, and the characteristic wavelengths were compressed to 71.6% of the total number of bands, which shortened the computation time; the characteristic bands were mainly located in the vicinity of 1050 nm, 1070–1280 nm, 1410–1500 nm, 1550–1580 nm, and 1550–1580 nm. The characteristic wavelength of the stretching vibration of the C-O bond was located near 1050 nm, corresponding to the absorption peaks of carbohydrate and alcohol compounds in buckwheat grains [40]. The peaks in the range of 1070–1280 nm correspond to the absorption of polysaccharides, proteins, and other compounds in buckwheat grains [41]. The peaks in the 1410–1500 nm wavelength range correspond to the absorption of flavonoids and other compounds in buckwheat seeds [42], and the 1550–1580 nm band may be related to the vibration of the C = C double bond [43].

## 5. Conclusions

(1) In this study, six buckwheat harvests (with 83-day, 90-day, 93-day, 96-day, 99-day and 102-day growth cycles) were investigated. The six different harvests of buckwheat kernels were analyzed using physicochemical tests to determine the buckwheat grain nutrient quality over the different harvest periods and the changes in buckwheat grain protein, fat, and starch contents. The total flavonoid and total phenol contents were higher in the 90-day cycle; thus, this was determined as the optimal buckwheat harvest period.
(2) In this study, near-infrared spectroscopy nondestructive testing technology was utilized to detect buckwheat content in six harvesting periods: 83 days, 90 days, 93 days, 96 days, 99 days, and 102 days. Through the comparison of six classification models, it was shown that the IVSO-RF model showed the best classification of different buckwheat harvests as the evaluation indexes were higher. When applying the IVSO-RF classification model to classify and validate a single buckwheat harvest period, the correctness rate of the training set for each harvest period reached 96%, and the correctness rate of the prediction set reached 100%.

In summary, through physical and chemical tests of the nutritional quality of buckwheat in different harvest periods, we determined that the optimal buckwheat harvest period is a growth cycle of 90 days. We employed near-infrared spectroscopy nondestructive testing technology combined with an IVSO-RF modeling method for buckwheat harvests to provide rapid, nondestructive classification and to determine the best harvest period. These physical and chemical tests, when combined with near-infrared spectroscopy, can determine the optimal period to achieve the best buckwheat harvest. The rapid study of different harvest periods, which lead to improvements in the nutritional quality of buckwheat, can also be applied to other crops to determine the optimal harvest period.

## Figures and Tables

**Table 1:** Content of essential nutrients in different harvest periods.

Harvest Period	Starch/%	Fat/%	Protein/%	Total Flavonoids (mg/g)	Total Phenols (mg/g)
83 days	47.12 ± 3.22 b	3.04 ± 0.46 ab	14.46 ± 0.46 a	4.23 ± 0.04 d	4.33 ± 0.04 b
90 days	55.66 ± 0.97 a	3.64 ± 0.23 a	14.76 ± 0.15 a	5.80 ± 0.06 ab	4.56 ± 0.02 a
93 days	52.49 ± 5.01 ab	3.29 ± 0.20 ab	14.60 ± 0.06 a	5.41 ± 0.11 c	4.32 ± 0.03 b
96 days	54.32 ± 1.90 a	3.04 ± 0.07 ab	14.73 ± 0.15 a	5.49 ± 0.04 c	4.33 ± 0.03 b
99 days	55.63 ± 0.17 a	2.78 ± 0.36 b	13.43 ± 0.06 b	5.39 ± 0.07 bc	4.11 ± 0.03 c
102 days	56.28 ± 3.17 a	2.65 ± 0.03 b	14.09 ± 0.20 ab	5.92 ± 0.06 a	4.23 ± 0.04 a
SD	3.44	0.36	0.51	0.60	0.15
Mean	53.58	3.07	14.35	5.37	4.31
CV	6.43%	11.60%	3.56%	11.18%	3.43%

Note: Different letters indicate significant differences (*p* < 0.05).

**Table 2:** Comparison of modeling results of RF and LS-SVM models based on different pretreatment methods.

Model	Preprocessing Method	Training Set Correctness (%)	Prediction Set Correctness (%)
RF	S-G	85.11	69.33
	SNV	94	92
	DWT	92.67	89
	normaliz	90.44	87.33
LS-SVM	S-G	40.67	36
	SNV	55.78	64.67
	DWT	42.89	44
	normaliz	44.22	42.67

**Table 3:** Comparison of prediction set results based on different classification models.

Model	Number	Total	NFP	Accuracy/%	Error Rate/%	Precision/%	Recall/%	F1 Score/%
IVSO-RF	106	150	5	96.67	3.33	96.62	89.16	92.58
VCPA-RF	14	150	25	83.33	16.67	83.94	81.23	82.99
VISSA-RF	77	150	7	95.33	4.67	95.54	89.03	92.43
IVSO-LS-SVM	106	150	79	47.33	52.67	54.94	66.95	44.31
VCPA-LS-SVM	14	150	103	31.33	68.67	37.58	29.18	27.60
VISSA-LS-SVM	77	150	81	46	54	53.99	50.25	41.87

Note: NFP, number of false positives.

## Data Availability

The original contributions presented in the study are included in the article, further inquiries can be directed to the corresponding author.
